# Pilot Study to Determine Accuracy of Posterior Approach Ultrasound for Shoulder Dislocation by Novice Sonographers

**DOI:** 10.5811/westjem.2016.2.29290

**Published:** 2016-04-26

**Authors:** Shadi Lahham, Brent Becker, Alan Chiem, Linda M. Joseph, Craig L. Anderson, Sean P. Wilson, Mohammad Subeh, Alex Trinh, Eric Viquez, John C. Fox

**Affiliations:** * University of California Irvine, Department of Emergency Medicine, Orange, California; † Wellspan York Hospital, Department of Emergency Medicine, York, Pennsylvania; ‡ Olive View-UCLA Medical Center, Department of Emergency Medicine, Los Angeles, California

## Abstract

**Introduction:**

The goal of this study was to investigate the efficacy of diagnosing shoulder dislocation using a single-view, posterior approach point-of-care ultrasound (POCUS) performed by undergraduate research students, and to establish the range of measured distance that discriminates dislocated shoulder from normal.

**Methods:**

We enrolled a prospective, convenience sample of adult patients presenting to the emergency department with acute shoulder pain following injury. Patients underwent ultrasonographic evaluation of possible shoulder dislocation comprising a single transverse view of the posterior shoulder and assessment of the relative positioning of the glenoid fossa and the humeral head. The sonographic measurement of the distance between these two anatomic structures was termed the Glenohumeral Separation Distance (GhSD). A positive GhSD represented a posterior position of the glenoid rim relative to the humeral head and a negative GhSD value represented an anterior position of the glenoid rim relative to the humeral head. We compared ultrasound (US) findings to conventional radiography to determine the optimum GhSD cutoff for the diagnosis of shoulder dislocation. Sensitivity, specificity, positive predictive value, and negative predictive value of the derived US method were calculated.

**Results:**

A total of 84 patients were enrolled and 19 (22.6%) demonstrated shoulder dislocation on conventional radiography, all of which were anterior. All confirmed dislocations had a negative measurement of the GhSD, while all patients with normal anatomic position had GhSD>0. This value represents an optimum GhSD cutoff of 0 for the diagnosis of (anterior) shoulder dislocation. This method demonstrated a sensitivity of 100% (95% CI [82.4–100]), specificity of 100% (95% CI [94.5–100]), positive predictive value of 100% (95% CI [82.4–100]), and negative predictive value of 100% (95% CI [94.5–100]).

**Conclusion:**

Our study suggests that a single, posterior-approach POCUS can diagnose anterior shoulder dislocation, and that this method can be employed by novice ultrasonographers, such as non-medical trainees, after a brief educational session. Further validation studies are necessary to confirm these findings.

## INTRODUCTION

Acute shoulder dislocation is common in the emergency department (ED) with an estimated incidence rate of 23.9 per 100,000 person-years.^1^ This affects 1.7% of the population and results in nearly 200,000 ED visits annually.^2^ Overall, 95–98% of shoulder dislocations are anterior. 3–5 Prompt recognition of this condition is essential for effective treatment, as reduction becomes increasingly difficult the longer the shoulder remains dislocated.^6^ The current standard approach for patients with suspected shoulder dislocation involves conventional radiography, performed both before and after reduction of the joint.^1^,^2^,^7^ Such imaging is often delayed in the ED due to a myriad of factors, prolonging patient discomfort and potentially complicating subsequent attempts at reduction.

Ultrasound (US) provides a rapid, point-of-care (POC) imaging modality that may facilitate the physician’s diagnosis of shoulder dislocation at the bedside and expedite definitive treatment.^3^,^4^,^8^ The orthopedic literature has demonstrated applicability of US in the evaluation of chronic shoulder conditions, such as instability, rotator cuff injuries and labral tears in the outpatient clinic setting. However, there is sparse literature documenting the use of US to diagnose acute shoulder injury or suspected dislocation.^9^–^13^ The emergency medicine literature contains several case reports detailing successful emergency physician (EP) use of US to evaluate shoulder dislocations. However, only one prospective study, by Abbasi et al, examines POCUS for the diagnosis of acute shoulder injuries.^8^,^14^,^15^,^16^ In the Abbasi study, all USs were performed by two sonographers, one of whom was particularly experienced in shoulder US, and entailed obtaining multiple sonographic views of the shoulder.^16^ A streamlined, single-view US technique to evaluate for shoulder dislocation that is accessible to even the most inexperienced sonographers would be of clinical benefit to EPs.

The primary objective of this study was to derive a standardized method for diagnosing shoulder dislocation using a single-view, posterior approach POCUS performed by undergraduate research students. A secondary objective was to determine the accuracy of this sonographic method in the diagnosis of shoulder dislocation by inexperienced sonographers.

## MATERIALS AND METHODS

### Study Design

This was a prospective, observational study of adult patients presenting with acute, traumatic shoulder pain aimed at deriving a single-view, sonographic method of diagnosing shoulder dislocation. This was based on the relative positioning of the glenoid fossa and the humeral head, as well as the distance between these two structures. Undergraduate research students enrolled all patients, performed the USs and calculated sonographic measurements. The study was approved by the study site institutional review board.

### Setting

The study was conducted at a single, urban, university-based hospital ED with an emergency medicine residency and an annual census of approximately 50,000 patients.

### Selection of Participants

All adult ED patients at least 18 years of age presenting with an acute, traumatic shoulder pain necessitating conventional radiography were eligible for inclusion. We excluded patients if they were under 18 years of age, not expected to undergo conventional radiography, incarcerated or could not otherwise provide consent.

A convenience sample of patients was enrolled between April 2011 and January 2015 daily between 8:00am and midnight. Undergraduate research students approached all adult ED patients presenting with a complaint of acute, traumatic shoulder pain for whom the treating physician ordered conventional radiographs of the shoulder. The students were present in the ED daily between 8:00am and midnight, but no patients were enrolled between the hours of midnight and 8:00am due to the lack of research staff availability. As part of the undergraduate research program, all participating students were fully trained in the consenting and enrollment process.

After providing written consent and before receiving conventional radiographs, enrolled patients underwent bedside ultrasonography of the affected shoulder. Undergraduate students with no previous sonographic experience collected all data. These students were enrolled in an emergency medicine research course and collected data for various studies. These novice sonographers were blinded to the results of the radiographs. While it was not possible to blind the undergraduate research students to the physical appearance of the shoulder, they did not possess formal medical training in anatomy or physical examination, which may have effectively blinded them from a clinical assessment. Similarly, conventional radiographs were formally read by attending radiologists blinded to the results of the POCUS.

### US Technique

Prior to the start of patient enrollment, each undergraduate research student received a 30-minute lecture on basic shoulder anatomy followed by a 30-minute hands-on US training session. This session was standardized and given by the US director at our institution. This was repeated once per year for a total of three sessions over the entire enrollment period.

**Figure 1:**
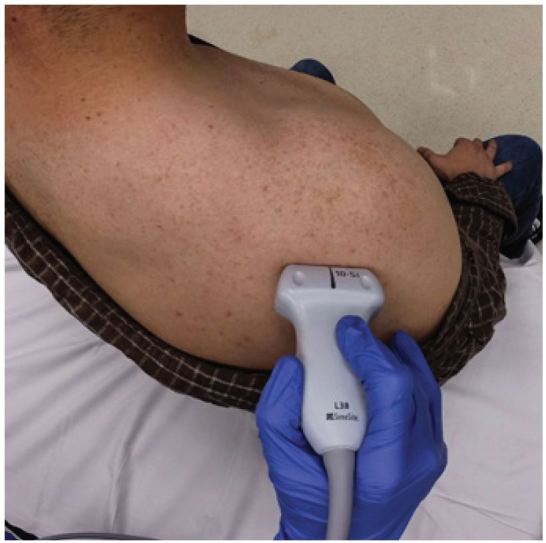
Superior view illustrating a posterior approach to a right shoulder ultrasound.

**Figure 2:**
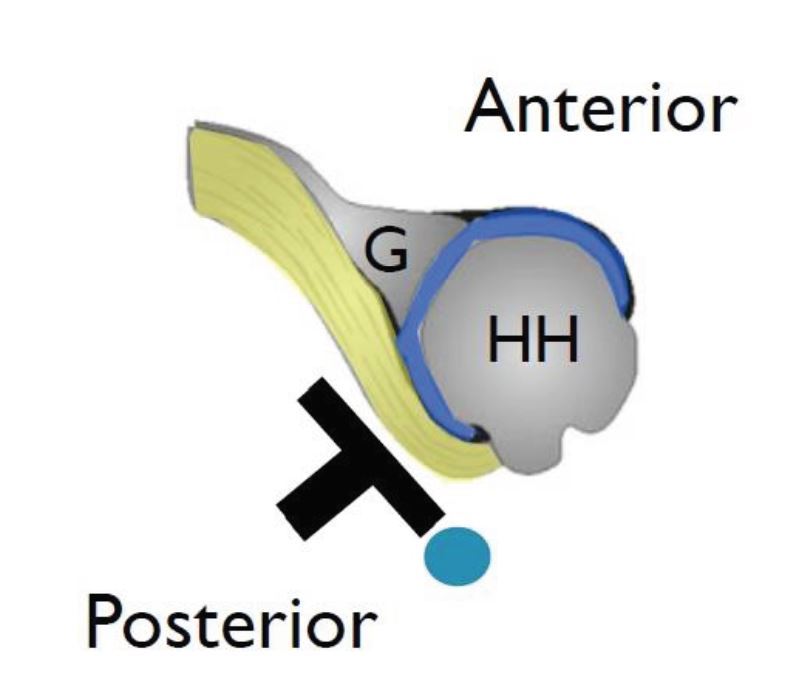
Schematic illustration demonstrating anatomic probe position of the right shoulder with an overhead view. *G*, glenoid; *HH*, humeral head

**Figure 3:**
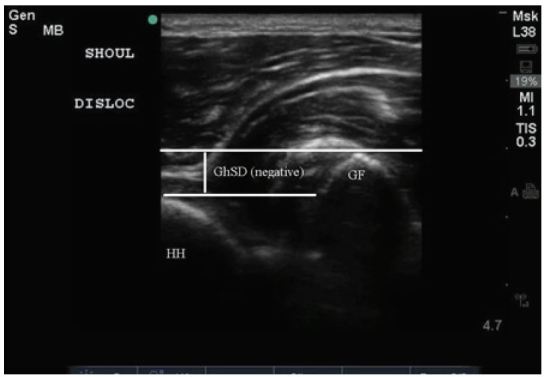
Ultrasound image depicting dislocated right shoulder. Both the humeral head (HH) and glenoid fossa (GF) are depicted with an illustration of the measured glenohumeral separation distance (GhSD).

**Figure 4:**
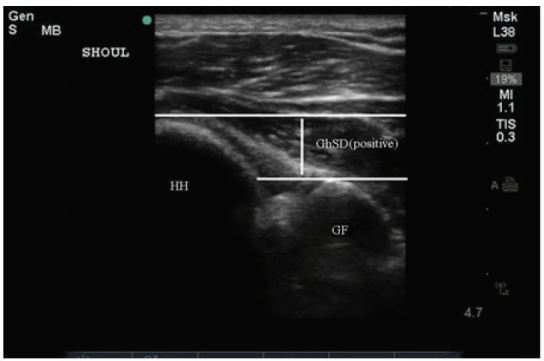
Ultrasound image depicting normal right shoulder anatomy. Both the humeral head (HH) and glenoid fossa (GF) are depicted with an illustration of the measured glenohumeral separation distance (GhSD).

Each undergraduate research student performed the US study using a Sonosite Edge (FUJIFILM Sonosite Inc). A 10–5 Mhz linear transducer was placed transversely on the posterior aspect of the patient’s shoulder with the probe indicator to the patient’s right with the patient seated upright. Both the glenoid rim and humeral head were visualized and identified (Figures 1, 2). For both the glenoid rim and the humeral head, the student operator placed a horizontal line, tangent to the most posterior aspect of each of the two anatomic structures. The distance between these two parallel lines was measured by a third line placed perpendicular to the previous two and defined the Glenohumeral Separation Distance (GhSD) (Figure 3, 4). The GhSD, measured in centimeters (cm), was given a positive or negative value based on the location of the glenoid rim relative to the humeral head. A positive GhSD value (GhSD>0cm) represented a posterior position of the glenoid rim relative to the humeral head and a negative GhSD value (GhSD<0cm) represented an anterior position of the glenoid rim relative to the humeral head. A GhSD value of 0cm implied that the most posterior aspect of the glenoid rim and humeral head were in perfect vertical alignment on the screen (Figures 3, 4). The GhSD values were measured in real time by the undergraduate students and recorded using standardized data collection forms.

### Outcome Measures

The primary sonographic outcome measure of interest was the GhSD. This measurement was correlated with the presence or absence of a dislocation seen on conventional radiography to derive an appropriate GhSD cutoff for diagnosing shoulder dislocation with US.

### Primary Data Analysis

Study data were entered in Excel (Microsoft, Redmond WA) and analyzed using Stata (version 12.1, StataCorp, College Station TX). We compared patient characteristics using chi-square tests for independence and t-tests, with calculation of exact binomial confidence intervals. The optimum GhSD cutoff value was determined to be used in defining the derived US method. Finally, we calculated the sensitivity, specificity, positive predictive value (PPV), and negative predictive value (NPV) of the POCUS method.

## RESULTS

**Figure 5:**
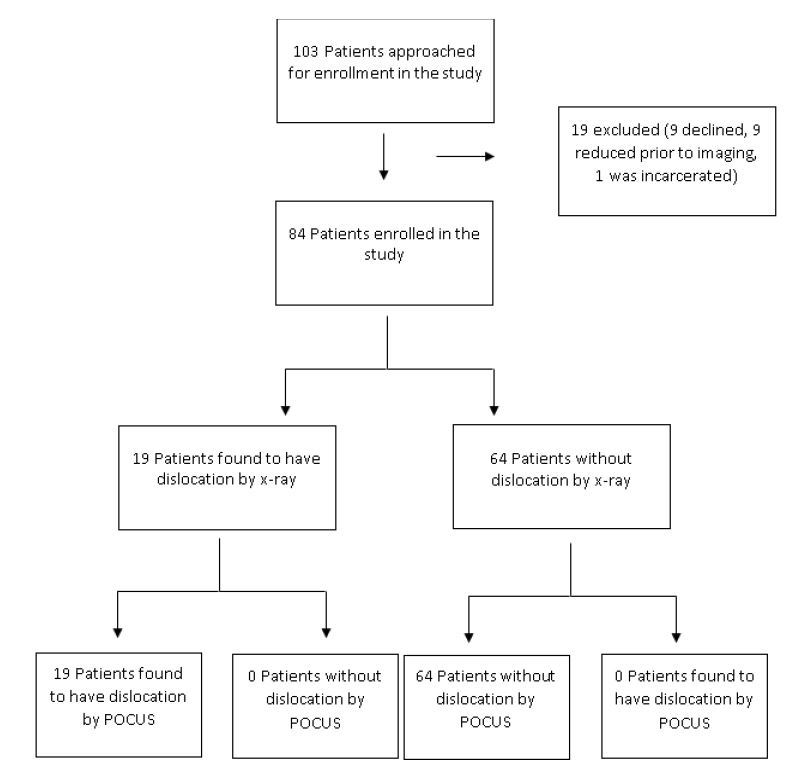
103 patients were screened for enrollment; 19 were ineligible and 84 patients consented and were enrolled. Of these, 19 had shoulder dislocations and 64 patients did not. *POCUS,* point of care ultrasound

A total of 103 patients were approached for enrollment in the study, of whom nine declined to participate and one was a prisoner. Nine patients were considered for enrollment but were diagnosed clinically and reduced at the bedside by provider prior to radiography. We included 84 patients in the data analysis, enrolled by 31 of 54 trained undergraduate research students (Figure 5). Patient ages ranged from 19 to 72 years old with a median age of 45 years old. Nineteen (22.6%) patients had radiography-confirmed dislocations, all of which were anterior. Fourteen of 52 male patients (26.9%) and 5 of 32 (19.6%) female patients had shoulder dislocations.

**Figure 6:**
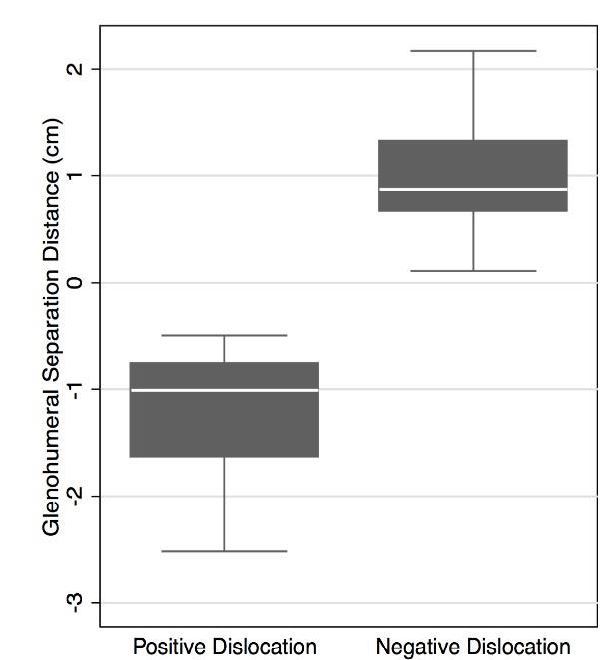
The measured glenohumeral separation of patients arranged by positive and negative dislocation.

The GhSD in patients with confirmed dislocations and in patients without dislocation are shown in Table 1 and Table 2, respectively. All confirmed dislocations demonstrated a GhSD<0cm and all confirmed non-dislocations demonstrated a GhSD>0cm. This is represented graphically in Figure 6. Thus GhSD=0cm was chosen as the cutoff value for the diagnosis of anterior shoulder dislocation. This derived POCUS method demonstrated a sensitivity of 100% (95% CI [82.4–100]), specificity of 100% (95% CI [94.5–100]), PPV of 100% (95% CI [82.4–100]), and NPV of 100% (95% CI [94.5–100]) in the diagnosis of anterior shoulder dislocations.

## DISCUSSION

Shoulder dislocations are commonly diagnosed and managed in the ED. Unfortunately, history and physical examination is often insufficient to definitively make the diagnosis.^1^,^17^ Radiographic imaging is therefore almost always employed prior to attempting reduction, as well as after reduction to confirm successful reduction. Radiographs are also used to determine whether the patient has developed common complications of shoulder dislocations, such as a Bankart lesion or Hill-Sachs lesion. Currently, typical care dictates that these patients obtain pre- and post-reduction imaging to confirm dislocation and that ED reduction is successful.^17^, ^18^ Waiting to obtain radiographs to diagnose shoulder dislocations has potential to delay definitive care. A study by Shuster et al has demonstrated that pre-reduction plain film radiographs increases ED length of stay by 29.6 minutes.^19^ Multiple case reports have found US useful in identifying acute dislocations.^8^,^14^,^15^,^20^ This is a promising imaging modality as not all healthcare facilities may have access to plain film imaging, especially in developing countries and austere environments.

US confers a number of potential benefits in the diagnosis and management of acute shoulder dislocation. It has been shown that sonographic diagnosis of shoulder dislocation can be obtained within five minutes of initial evaluation.^16^ This efficiency is especially helpful in patients requiring procedural sedation for reduction. An unsuccessful reduction may necessitate repeat sedation if a patient recovers before imaging can be performed.^21^ Therefore, POCUS may be used to confirm adequate shoulder reduction, which could eliminate the need for repeat sedation and its associated risks. There is also the potential to decrease radiation exposure, especially in patients who require multiple manipulations.

A recent study by Abbasi et al, employing a combined anterior and lateral approach, showed a sensitivity and specificity of 100% for US in the diagnosis of shoulder dislocation in 69 dislocated patients among 73 studied.^16^ The study demonstrated no difference in image acquisition and interpretation between an experienced emergency sonographer and a senior emergency medicine resident who had received a one-hour lecture and performed 10 supervised shoulder USs. This study suggests that physicians with focused training are able to accurately identify shoulder dislocations using POCUS; however, it remains unclear if non-medically trained practitioners can do the same.

In our study, we attempted to derive a standardized method for diagnosing shoulder dislocation using a single-view, posterior approach POCUS technique. Given that there is no way to blind clinicians from physical exam findings and to eliminate the possibility of physical exam bias, undergraduate research students without formal anatomy or medical training enrolled patients and performed USs. While there are several physical exam findings indicative of shoulder dislocation, there are no sonographic findings that are universally agreed upon.^15^

We propose the following method of diagnosing anterior shoulder dislocations using a posterior transverse US approach: A positive humeral head distance relative to the glenoid indicates a normally placed shoulder, while a negative humeral head distance relative to the glenoid indicates an anteriorly dislocated shoulder. Use of this measurement for the diagnosis of anterior shoulder dislocation resulted in a sensitivity of 100%, and specificity of 100%, with no overlap in the GhSD between the dislocated and non-dislocated groups (Figure 5).

Our data suggest that despite minimal training, image acquisition and measurements obtained by non-clinical personnel were found to be adequate for clinical use. More importantly, given the large number of true negatives, we hope to use these measurements to help create a standard approach to shoulder US US and standardize a sonographic definition of a dislocated shoulder. While these results may not be generalizable to all ED clinicians, they do show promise in the sense that with minimal training, clinicians can potentially obtain images that can be used for clinical decision-making. Furthermore, given their lack of clinical training, these students were effectively blinded from making a clinical diagnosis of shoulder dislocation. Given the strong correlation of the direction of the humeral head relative to the glenoid to the presence of a shoulder dislocation, we believe that clinicians can employ this technique with minimal training to expedite the diagnosis of anterior shoulder dislocation.

## LIMITATIONS

There are several limitations to our study. We used a convenience sample that was small and enrollment was limited to a single center. Nine patients were reduced prior to enrollment without additional imaging. We were also unable to blind sonographers to the visual appearance of the shoulder and potential deformities. US scans were not repeated by more experienced practitioners to confirm findings. Data on injuries other than glenohumeral dislocation, such as fractures, were not specifically collected and the ability of US to detect other potential shoulder injuries was not evaluated. No posterior dislocations were diagnosed during the course of the study. While posterior dislocations are rather uncommon, we cannot comment on the ability of this technique to diagnosis a posterior shoulder dislocation. Given glenohumeral anatomy, posterior shoulder ultrasonography may not be able to correctly identify a posterior dislocation.

## CONCLUSION

Our findings suggest that a single, posterior-approach POCUS technique can diagnose anterior shoulder dislocation and can be employed by novice, non-medical trainees after a brief educational session. We propose a definition that using a posterior transverse US of the shoulder, a positive humeral head distance relative to the glenoid indicates a normally placed shoulder while a negative step off distance of the humeral head relative to the glenoid indicates an anteriorly dislocated shoulder. Further validation studies are necessary to confirm these findings.

## References

[1] Zacchilli MA, Owens BD (2010). Epidemiology of shoulder dislocations presenting to emergency departments in the United States. J Bone Joint Surg Am.

[2] Youm T, Takemoto R, Park BKH (2014). Acute Management of Shoulder Dislocations. J Am Acad Orthop Surg.

[3] Davids JR, Talbott RD (1990). Luxatio erecta humeri. A case report. Clin Orthop Relat Res.

[4] Yamamoto T, Yoshiya S, Kurosaka M (2003). Luxatio erecta (inferior dislocation of the shoulder): a report of 5 cases and a review of the literature. Am J Orthop (Belle Mead NJ).

[5] Westin CD, Gill EA, Noyes ME (1995). Anterior shoulder dislocation. A simple and rapid method for reduction. Am J Sports Med.

[6] Allen G (2008). Shoulder ultrasound imaging – integrating anatomy, biomechanics and disease process. Eur J Radiol.

[7] McNamara R, Roberts JR, Hedges JR (1998). Management of common dislocations. Clinical procedures in emergency medicine.

[8] Halberg M, Sweeney TW, Owens WB (2009). Bedside ultrasound for verification of shoulder reduction. Am J Emerg Med.

[9] Schydlowsky P, Strandberg C, Galbo H (1998). The value of ultrasonography in the diagnosis of labral lesions in patients with anterior shoulder dislocation. Eur J Ultrasound.

[10] Kolla S, Motamedi K (2007). Ultrasound evaluation of the shoulder. Semin Musculoskelet Radiol.

[11] Morag Y, Jacobson JA, Lucas D (2006). US appearance of the rotator cable with histologic correlation: preliminary results. Radiology.

[12] Lee JC, Sykes C, Saifuddin A (2005). Adhesive capsulitis: sonographic changes in the rotator cuff interval with arthroscopic correlation. Skeletal Radiol.

[13] Jacobson JA, Lancaster S, Prasad A (2004). Full-thickness and partial-thickness supraspinatus tendon tears: value of US signs in diagnosis. Radiology.

[14] Blakeley CJ, Spencer O, Newman-Saunders T (2009). A novel use of portable ultrasound in the management of shoulder dislocation. Emerg Med J.

[15] Yuen CK, Mok KL, Kan PG (2009). Bedside ultrasound for verification of shoulder reduction with the lateral and anterior approaches. Am J Emerg Med.

[16] Abbasi S, Molaie H, Hafezimoghadam P (2013). Diagnostic accuracy of ultrasonographic examination in the management of shoulder dislocation in the emergency department. Ann Emerg Med.

[17] Hendey G (2000). Necessity of radiographs in the emergency department management of shoulder dislocations. Ann Emerg Med.

[18] Riebel GD, McCabe JB (1991). Anterior shoulder dislocation: a review of reduction techniques. Am J Emerg Med.

[19] Shuster M, Abu-Laban RB, Boyd J (1999). Prereduction radiographs in clinically evident anterior shoulder dislocation. Am J Emerg Med.

[20] Stone M, Sutijono D (2009). Dynamic Emergency Medicine: Intraarticular injection and closed glenohumeral reduction with emergency ultrasound. Acad Emerg Med.

[21] Wen DY (1999). Current concepts in the treatment of anterior shoulder dislocations. Am J Emerg Med.

